# Three-Dimensional Biofabrication Models of Endometriosis and the Endometriotic Microenvironment

**DOI:** 10.3390/biomedicines8110525

**Published:** 2020-11-21

**Authors:** Jillian R. H. Wendel, Xiyin Wang, Lester J. Smith, Shannon M. Hawkins

**Affiliations:** 1 Department of Obstetrics and Gynecology, Indiana University School of Medicine, Indianapolis, IN 46202, USA; jhufgard@iu.edu (J.R.H.W.); xw49@iu.edu (X.W.); 2 Department of Radiology and Imaging Sciences, Indiana University School of Medicine, Indianapolis, IN 46202, USA; smitlej@iu.edu; 3 3D Bioprinting Core, Indiana University School of Medicine, Indianapolis, IN 46202, USA; 4 Department of Biochemistry and Molecular Biology, Indiana University School of Medicine, Indianapolis, IN 46202, USA; 5 Indiana University Melvin and Bren Simon Comprehensive Cancer Center, Indiana University School of Medicine, Indianapolis, IN 46202, USA

**Keywords:** endometriosis, 12Z, spheroid, biofabrication, scaffold-free, Kenzan

## Abstract

Endometriosis occurs when endometrial-like tissue grows outside the uterine cavity, leading to pelvic pain, infertility, and increased risk of ovarian cancer. The present study describes the optimization and characterization of cellular spheroids as building blocks for Kenzan scaffold-free method biofabrication and proof-of-concept models of endometriosis and the endometriotic microenvironment. The spheroid building blocks must be of a specific diameter (~500 μm), compact, round, and smooth to withstand Kenzan biofabrication. Under optimized spheroid conditions for biofabrication, the endometriotic epithelial-like cell line, 12Z, expressed high levels of estrogen-related genes and secreted high amounts of endometriotic inflammatory factors that were independent of TNFα stimulation. Heterotypic spheroids, composed of 12Z and T-HESC, an immortalized endometrial stromal cell line, self-assembled into a biologically relevant pattern, consisting of epithelial cells on the outside of the spheroids and stromal cells in the core. 12Z spheroids were biofabricated into large three-dimensional constructs alone, with HEYA8 spheroids, or as heterotypic spheroids with T-HESC. These three-dimensional biofabricated constructs containing multiple monotypic or heterotypic spheroids represent the first scaffold-free biofabricated in vitro models of endometriosis and the endometriotic microenvironment. These efficient and innovative models will allow us to study the complex interactions of multiple cell types within a biologically relevant microenvironment.

## 1. Introduction

Endometriosis is a common disease affecting 178–200 million women and girls worldwide [1,2,3]. Endometriosis occurs when endometrial-like tissue grows outside the uterine cavity, frequently invading structures within the pelvic cavity, including the peritoneum, ovaries, bladder, small intestine, or colon. Women with endometriosis can exhibit debilitating chronic pelvic pain and infertility [1,2,3]. Endometriosis often results in decreased quality of life, as measured by the Endometriosis Health Profile-30 (EHP-30) [4,5,6,7,8]. Women with endometriosis have higher hospital admissions rates, increased work and school day absences, and increased economic burden [9,10,11]. Women with endometriosis are also at a significantly increased risk for developing ovarian cancer, specifically the endometriosis-associated ovarian cancers, including clear cell or endometrioid ovarian adenocarcinoma [12,13,14,15].

The scarcity of representative endometriotic in vitro models hinders research within the endometriosis field [16,17,18,19]. Immortalized stromal cell lines have been developed from endometrial tissue from women with endometriosis or endometriotic lesions [20,21]. However, few immortalized epithelial endometriotic cell lines exist, with 12Z cells being the most studied [16,21,22]. While traditional monolayer culture has set the groundwork for many advancements, limitations are seen in its inability to recapitulate therapeutic outcomes in vivo [23]. These limitations may be due to the lack of reflection of the complete and complex three-dimensional (3D) interactions and their surrounding microenvironment [17]. For example, 3D models better recapitulate the highly inflammatory microenvironment of endometriosis compared to monolayer [24]. Additionally, 3D models of ovarian cancer better represent human tissue characteristics, gene expression patterns, and chemotherapy response [24,25,26,27,28]. However, for in vitro models to better represent endometriosis and the endometriotic microenvironment, continued advancement is required.

In the present study, we describe a method of culturing spheroids (~500 μm) that retain endometriotic characteristics. Additionally, we provide proof-of-concept models of complex 3D biofabricated tissue-like constructs using the scaffold-free Kenzan method [29] to assemble spheroids into larger constructs. This body of work is intended as a proof-of-concept foundation to use these modalities to study endometriosis and the endometriotic microenvironment.

## 2. Experimental Section

### 2.1. Biofabrication Equipment and the Kenzan Method

**Figure 1:**
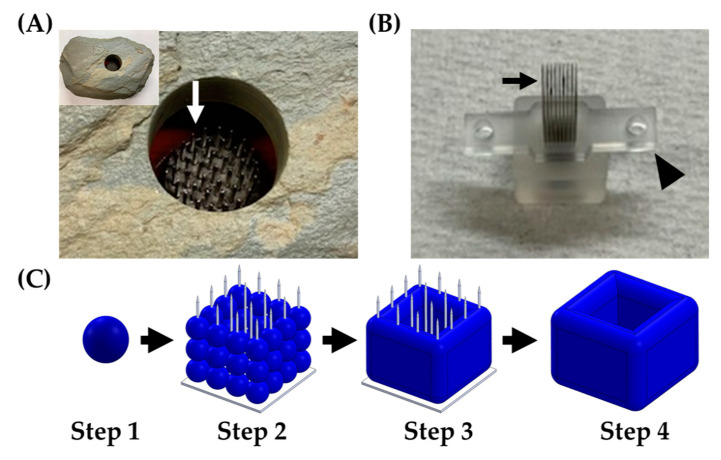
Kenzan method of biofabrication. (**A**) A kenzan (Ansel Adams Museum, Yosemite National Park, CA, USA) used for flower arranging. Needles (white arrow) would hold the floral stems in place. (**B**) A Kenzan used for 3D biofabrication with the Regenova Bio 3D Printer. This Kenzan is made of a 9 × 9 stainless steel needle array (black arrow) and supported by two platens (arrowhead) to hold the needles in alignment and aid in removing the construct. (**C**) Workflow of Kenzan method biofabrication. Step 1—Generation of building blocks for biofabrication, called spheroids, large (~500 μm), tight, dense masses of cells that do not break up with gentle disruption. Step 2—Spheroids are placed onto the needles of the Kenzan with a robotic arm’s assistance. The X, Y, and Z placement on Kenzan is user-defined, allowing for a wide range of construct designs. Step 3—Spheroids are incubated in media on the Kenzan, allowing for a naturally secreted extracellular matrix to fuse adjacent spheroids. Step 4—The Kenzan is gently removed with one of the two platens’ aid, leaving behind a scaffold-free construct.

The Regenova Bio 3D Printer (Cyfuse Biomedical K.K., Tokyo, Japan), a semi-automated biofabrication robot, was used in two distinct ways for the scope of this work. First, the Regenova Bio 3D Printer used a vision system to examine the spheroids and assess the roundness, surface smoothness, and diameter [29,30,31]. This assessment was used to optimize the spheroid parameters before biofabrication because the Kenzan method requires a specific set of characteristics for successful biofabrication. Second, the Regenova Bio 3D Printer used spheroids as building blocks for 3D biofabrication using the Kenzan method. The Kenzan method of biofabrication is conceptualized from the Japanese Ikebana method of flower arranging. In the Japanese form of flower arrangement, a kenzan is a metal needle array used to hold floral arrangements in place (Figure 1A). In the Kenzan method of biofabrication, the Kenzan is the microneedle array (Figure 1B) used to hold cell spheroids [29,30,31] in contact with one another so they can fuse to form larger, more sophisticated tissue constructs. The Kenzan (Amuza, Inc., San Diego, CA, USA) used was a 9 × 9 microneedle array (width: 10 mm, depth: 20 mm, height: 18 mm) made from stainless steel and housed in a biocompatible casing containing phosphate-buffered saline. The Regenova Bio 3D Printer interfaced with the Bio 3D Designer software (Cyfuse Biomedical K.K.) to delineate the user-defined construct design. The robotic arm of the Regenova lifted the spheroids from the 96-well plate, using a vacuum nozzle (Amuza, Inc.), and placed each spheroid on the Kenzan in a user-defined pattern. The Kenzan containing the biofabricated spheroids was transferred to a culture dish containing media and incubated in a humidified incubator at 37 °C and 5% CO_2_, allowing the spheroids to fuse into a larger tissue construct. The construct was removed from the Kenzan, leaving behind an utterly scaffold-free construct [29] comprised only of cells and their self-secreted extracellular matrix (Figure 1C). The parameters used are reported below.

### 2.2. Cell Lines

12Z cells, a SV40-transformed endometriotic epithelial-like cell line derived from red peritoneal endometriosis lesions [21], were obtained from Asgi Fazleabas, Ph.D. (Michigan State University College of Human Medicine, Grand Rapids, MI, USA) with written permission from Anna Starzinski-Powitz, Ph.D. (Wolfgang Goethe Frankfurt University, Frankfurt, Germany). 12Z cells were maintained in Dulbecco’s modified Eagle media (DMEM)/F12 (Thermo Fisher Scientific, Waltham, MA, USA), supplemented with 10% fetal bovine serum [(FBS), Atlanta Biologicals, Minneapolis, MN, USA or Corning Inc, Corning, NY, USA] and 1% penicillin/streptomycin [(P/S), Thermo Fisher Scientific]. HEYA8, an epithelial ovarian cancer cell line, established from murine peritoneal tumors, following injection with the parent HEY cell line [32], was obtained from the Cytogenetics and Cell Authentication Core at the University of Texas MD Anderson Cancer Center (Houston, TX, USA). HEYA8 cells were maintained in RPMI (Thermo Fisher Scientific), supplemented with 10% FBS (Atlanta Biologicals or Corning) and 1% P/S. T-HESC, a human telomerase-transformed endometrial stromal cell line [33], was obtained through American Type Culture Collection (ATCC) (Manassas, VA, USA, CRL-4003). T-HESCs were maintained in DMEM/F12 without phenol red (Thermo Fisher Scientific), supplemented with 10% FBS (Atlanta Biologicals), 1% Corning ITS^TM^+ Premix Universal Culture Supplement [insulin (5 μg/mL), transferrin (5 μg/mL), and selenious acid (5 ng/mL) (Thermo Fisher Scientific)], and 500 ng/mL puromycin (Thermo Fisher Scientific). HEK293T, an adenovirus type 5 DNA-transformed human embryonic kidney cell line [34], was obtained through American Type Culture Collection (ATCC, CRL-3216). HEK293T cells were maintained in DMEM (Thermo Fisher Scientific) supplemented with 10% FBS (Atlanta Biologicals) and 1% P/S. All cell lines were maintained in a humidified incubator at 37 °C and 5% CO_2_. Cell line authentication was confirmed using CellCheck 9 Plus (IDEXX BioAnalytics, Westbrook, ME, USA). All cell lines were negative for mycoplasma contamination (MycoAlert Plus Mycoplasma Detection Kit, Lonza, Switzerland).

### 2.3. Fluorescent Labeling of Live Cells

12Z cells were transfected with a Td-tomato marker. The tdTomato-C1 plasmid was a gift from Michael Davidson (Addgene plasmid #54653: http://n2t.net/addgene:54653; RRID: Addgene_54653). One day before transfection, 500,000 12Z cells per well were seeded in a 6-well plate. Lipid-based transfection (Lipofectamine^®^ 2000, Thermo Fisher Scientific) was carried out as per manufacturer’s instructions, using 2.5 µg DNA in triplicate. The following day cells were trypsinized, and triplicates were pooled and reseeded to three 10-cm dishes at 4,000,000 cells per plate. Cells were selected in 300 µg/mL of geneticin (Thermo Fisher Scientific). Fluorescent expression was confirmed on an EVOS FL Cell Imaging System (Thermo Fisher Scientific) using the EVOS RFP light cube (Thermo Fisher Scientific, excitation: 531/40 nm and emission: 593/40 nm).

HEYA8 and T-HESC cells were transduced to express a green fluorescent protein (GFP). Lentiviral shRNA particles were generated using HEK293T cells. HEK293T cells were seeded at 500,000 cells per well. Lipid-based transfection (Lipofectamine^®^ 2000) was carried out as per the manufacturer’s instructions. The GIPZ non-silencing control plasmid containing GFP was obtained from the Core Based Assay Screening Service (C-BASS) Core of Baylor College of Medicine. The lentiviral packaging mix (Rev1b, Tat1b, HGPM2, and VSVG) was mixed with the lipid media and added to the HEK293T cells, gently swirled, and incubated [35]. Media was changed at 24 h, and viral particles were collected at 48 and 72 h by removing media and spinning at 4 °C at 800 × *g* for 10 min. For transduction, 150,000 HEYA8 cells/well or 500,000 T-HESC cells/well were seeded. Transduction occurred via centrifugation with 5 µg/mL of polybrene (Sigma, St. Louis, MO, USA) at 800 × *g* for 60 min at room temperature followed by six hours of incubation. A volume of 0.5 mL of cell media was added to the cells and incubated overnight. The following day, media was replaced with fresh media. On day five post-transduction, HEYA8 cell media was supplemented with 1 µg/mL puromycin for selection. HEYA8 cells were maintained under selective pressure for two weeks. Fluorescence was confirmed on an EVOS FL Cell Imaging System using the EVOS GFP light cube (Thermo Fisher Scientific, excitation: 470/22 nm and emission: 525/50 nm). On day three post-transduction, T-HESC cells were seeded as a single cell per well. Fluorescence was confirmed, and the brightest colonies were expanded.

### 2.4. Optimization of Spheroids for Kenzan Biofabrication

Cell density, time in culture, and serum effects were assessed to determine the best conditions for spheroids for Kenzan biofabrication. Cells were seeded in PrimeSurface^®^ 3D Culture Spheroid plates: Ultra-low Attachment Plates (S-Bio, Hudson, NH, USA) and allowed to aggregate into spheroids over the course of up to 120 h. Spheroids were scanned using the Regenova Bio 3D Printer vision system daily for up to 5 consecutive days to assess the roundness, smoothness, and diameter. The Regenova Bio 3D designer software utilizes previously published equations to define roundness (%), smoothness (%), and diameter (µm) [30]. Biologically, we classified masses of cells as “spheroids” if gentle disruption by pipetting failed to break up the tight, dense mass. Optimal goal parameters for successful biofabrication were 80–100 roundness (%), 0–5 smoothness (%), and 450–650 diameter (µm), based on spheroids, which were successfully biofabricated in the past [30].

### 2.5. Scaffold-Free 3D Biofabrication Optimization

Data and tissue-like 3D biofabricated constructs were acquired at the 3D BioPrinting Core. Spheroids were 3D biofabricated onto a Kenzan in a user-defined 3D design (shown below) using the Regenova Bio 3D Printer [29,31]. Briefly, spheroids in 96-well ultra-low attachment round-bottom plates (S-Bio) were digitally scanned for roundness, smoothness, and diameter. If 80–100 roundness (%), 0–5 smoothness (%), and 450–650 diameter (µm) were met, the spheroid was picked up via 2 kPa of suction with a 26-gauge nozzle (Amuza, Inc.) and placed on the Kenzan via the robotic arm. Following biofabrication, spheroids on the Kenzan were incubated in media in a humidified incubator at 37 °C and 5% CO_2_ for 48–72 h. Constructs were removed from the Kenzan and incubated. To prevent adhesion to culture dishes, constructs were incubated in ultra-low attachment 12-well plates (Sigma).

### 2.6. Fixation of Spheroids and Constructs

Spheroids (five days post-seeding) or constructs (24 h after removal from the Kenzan) were collected, washed with 1X phosphate-buffered saline (PBS), fixed in 1% paraformaldehyde (Thermo Fisher Scientific) prepared in 1X PBS for 30 min at 4 °C, and washed in 1X PBS, followed by 0.85% sodium chloride in 1X PBS, and 0.85% sodium chloride in 70% ethanol for 30 min apiece at room temperature with gentle agitation. Spheroids and constructs were stored in 70% ethanol until embedding. For embedding, spheroids were placed at the bottom of a 15 mL tube, and 20 µL of pre-warmed HistoGel (Thermo Fisher Scientific) was added. The gel was allowed to solidify for 20 min at 4 °C. The plug was then removed, rinsed in 70% ethanol, and stored in 70% ethanol. Plugs and constructs were transferred to the Histology Core of the Indiana Center for Musculoskeletal Health at IU School of Medicine for processing, paraffin embedding, and 5 µm sectioning.

### 2.7. Immunofluorescent Staining

Paraffin was dissolved from 5 µm sectioned tissues, and tissue was rehydrated by a series of washes 3 min each: xylene, xylene, 1:1 xylene/100% ethanol, 100% ethanol, 100% ethanol, 95% ethanol, 70% ethanol, 50% ethanol, and five minutes in water. Heat-mediated antigen retrieval was performed by submerging slides in pre-warmed citrate buffer, pH 6.0 (Vector Laboratories, Burlingame, CA, USA) for three minutes on high in a pressure cooker (Nesco, Two Rivers, WI, USA) followed by four minutes on warm. Slides were allowed to cool to room temperature before being washed with gentle agitation for three minutes each: water, tris-buffered saline (TBS), and TBS with 0.1% Tween 20 (TBS-T) (Thermo Fisher Scientific). Sections were permeabilized for nuclear detection via 0.4% Triton X-100 (Sigma) in 1X TBS for 10 min with gentle rocking followed by three TBS-T washes for three minutes each. Sections were blocked with 5% normal donkey serum (Jackson ImmunoResearch Laboratories, INC., West Grove, PA, USA) for one hour at room temperature in a humidified chamber. Sections were then washed with TBS-T three times for three minutes each with gentle rocking. Staining was completed on four independent spheroids for proliferation and apoptosis markers. Primary antibodies [Ki-67 (Millipore Sigma, AB9260, 1:50) and cleaved caspase 3 (cCAS3, Cell Signaling Technology, Danvers, MA, USA, 9661S, 1:100)] were diluted in 1% normal donkey serum. Sections were incubated overnight at 4 °C in a humidified chamber with the primary antibody. Slides were washed three times with TBS-T for three minutes with gentle rocking. The secondary antibody was diluted in 1% normal donkey serum (Donkey anti-Rabbit 555, Thermo Fisher Scientific, A31572, 1:100), and sections were incubated at room temperature for 2 h. Sections were then washed three times with TBS-T for three minutes with gentle rocking. The sections were mounted with Fluoromount-G mounting media with 4′,6-diamidino-2-phenylindole (DAPI, Thermo Fisher Scientific). Secondary only stained sections were used as a negative control. Immunofluorescent staining was imaged on an EVOS FL Cell Imaging System.

### 2.8. Immunohistochemical Staining

Section preparation methods for immunohistochemical staining of biofabricated constructs were similar to immunofluorescent staining of spheroids. Briefly, 5 µm sections were deparaffinized and rehydrated using a graded alcohol series. Rehydration was followed by heat-mediated antigen retrieval in citrate buffer, permeabilization in 0.4% Triton X-100, and quenching with 3% hydrogen peroxide (Thermo Fisher Scientific) in methanol (Thermo Fisher Scientific). Sections were blocked in 5% normal goat serum (Vector Laboratories) for 1 h. Sections were then washed with TBS-T three times for three minutes each with gentle rocking. Sections were incubated overnight with the primary antibody in a humidified chamber at 4 °C. Primary antibodies were diluted in 1% normal goat serum. Cytokeratin 7 (KRT7, Abcam, United Kingdom, Ab181598, 1:100) and membrane metalloendopeptidase (CD10, Abcam, Ab208778, 1:20,00) were used. Sections were washed and incubated at room temperature for one hour with goat-anti-rabbit horseradish peroxidase-conjugated secondary antibody (2 µg/mL, Vector Laboratories). Immunoreactivity was detected using the VECTASTAIN Elite ABC-Peroxidase Kit (Vector Laboratories) and the DAB Substrate Kit (Vector Laboratories) following the manufacturer’s instructions. Sections were then counterstained by incubating in hematoxylin (Sigma) for one min, rinsed in water, dipped in 0.025% hydrochloric acid (Thermo Fisher Scientific), rinsed in water, dipped in saturated lithium carbonate (Thermo Fisher Scientific) until nuclei turned blue, and then washed in water for five minutes with gentle agitation. Dehydration of sections was done by graded alcohol series: 50% ethanol for five minutes, 70% ethanol for 5 min, 80% ethanol for five minutes, 95% ethanol for 5 min, 100% ethanol for 10 min two times, and xylene for 10 min two times. Following dehydration, sections were mounted using mounting media (Thermo Fisher Scientific). Immunohistochemical staining was imaged on a Zeiss Axio Lab.A1 microscope (Zeiss, Oberkochen, Germany).

### 2.9. Secreted Inflammatory Cytokines Assessment

Monolayer cells were seeded 24 h, and spheroids were seeded 72 h before tumor necrosis factor-alpha (TNFα) stimulation. Both monolayer and spheroids were seeded with 10,000 cells. The media was replaced with serum-free media containing 15 ng/mL TNFα (Sigma) or an equal volume of dimethyl sulfoxide (DMSO, Thermo Fisher Scientific). Cells in monolayer and spheroid were incubated for 24 h before conditioned media collection. Conditioned media was collected and diluted 1:4, based on optimization (data not shown), with serum-free media and delivered to the Multiplex Analysis Core at the Indiana University Melvin and Bren Simon Comprehensive Cancer Center for analysis. Interleukin (IL)-6, IL-8, and monocyte chemotactic protein-1 (MCP1) were examined using the Human Cell Culture Supernatant 3-Plex Human Adipocyte Magnetic Bead Panel (HADCYMAG-61K, EMD Millipore, Burlington, MA, USA) following manufacturer’s instructions on the BioPlex 200 System (Bio-Rad, Hercules, CA, USA). A standard curve was generated based on the manufacturer’s instructions, and the kit supplied standards were used to determine the pg/mL of samples. Technical replicates were run in duplicate with 3–4 biological replicates.

### 2.10. RNA Extraction and qRT-PCR

In an independent experiment, wells were seeded with 20,000 cells per well for both monolayer and spheroid groups. RNA was extracted using the TaqMan MicroRNA Cells-to-CT Kit (Thermo Fisher Scientific) following the manufacturer’s instructions. A NanoDrop ND-1000 (Thermo Scientific) was used for nucleic acid quantification, and 1000 ng of RNA was reverse transcribed in a 20 µL reaction using 50 units of MultiScribe™ Reverse Transcriptase (RT, Thermo Fisher Scientific), 1X RT Buffer (Thermo Fisher Scientific), 5 mM deoxynucleoside triphosphate (Thermo Fisher Scientific), 2.5 µM random hexamers (Thermo Fisher Scientific), and 6 units of RNase inhibitor (Thermo Fisher Scientific). cDNA was generated in a 2720 Thermal Cycler (Thermo Fisher Scientific) with the following conditions: 10 min at 25 °C, 30 min at 48 °C, and 5 min at 95 °C. Samples were diluted to 100 µl and used for quantitative PCR (qPCR). Real-time qPCR (qRT-PCR) was performed using either previously published SYBR green primers [β-Actin [36]; cytochrome p450, family 19a1 (*CYP19A1*) [37]; or hydroxysteroid 17-beta dehydrogenase1 (*HSD17β1*) [37]] or predesigned TaqMan Gene Expression Assays [Thermo Fisher Scientific, estrogen receptor type 1 (*ESR1*) (Hs01046818_m1)]. β-Actin was previously used as an endogenous control in 12Z cells [36]. Additionally, no statistical difference (*p* > 0.1) was found in β-Actin when comparing the 2^−ΔCT^ across groups, and the cycle threshold (Ct) of each sample had a Z-Score of 1 or less compared to the whole. TaqMan PCR was performed using TaqMan Universal PCR Master Mix II, No UNG (Thermo Fisher Scientific). SYBR Green PCR was performed using SYBR Green PCR Master Mix (Thermo Fisher Scientific). Reaction conditions followed the recommendations on the QuantStudio3 Real-Time PCR System (Thermo Fisher Scientific). Each sample was run in duplicate, and a non-template control (nuclease-free water) was included in each plate. All SYBR green runs had dissociation curves to identify the potential primer-dimers. Relative quantification was determined using 2^−ΔΔCT^ [38]. One sample was excluded for being two standard deviations from the average, leaving 3–6 biological replicates per group.

### 2.11. Statistical Analysis

Statistical analyses were conducted through the InStat package for Prism8 (GraphPad, San Diego, CA, USA). Comparisons of two groups were performed using a two-sided Student’s *t*-test. A two-way ANOVA was used with Tukey’s multiple comparison test when more than two groups were present. Analysis of both treatment and gene/protein was performed using a two-way ANOVA with a Śidâk’s correction for multiple comparisons across treatment [39]. Data were presented as the mean and standard error of the mean. Significant differences between groups were determined by *p* < 0.05.

## 3. Results

### 3.1. 12Z Cells Grow as Large Spheroids within Parameters of Biofabrication by the Kenzan Method

The goal of this project was the 3D biofabrication of endometriosis and endometriotic microenvironment models using the Kenzan method. The first step of this process involved the production of spheroids within appropriate parameters for biofabrication on Kenzan. Based on spheroids successfully biofabricated in the past [30], optimal goal parameters for successful biofabrication were 80–100 roundness (%), 0–5 smoothness (%), and 450–650 diameter (µm). We started with 12Z cells because (1) 12Z cells were derived from a red peritoneal endometriosis lesion and transformed using SV40 T-antigen [21], allowing for a replenishable supply of cells; (2) the 12Z cells are commercially available (https://www.abmgood.com/immortalized-human-endometriotic-cell-line-12z.html) and have a reasonable doubling time [21] for efficient use in real-time experiments; (3) 12Z cells exhibit epithelial-like properties both in cytokeratin expression and morphology [21]; (4) 12Z cells exhibit high expression of prostaglandin E_2_, matrix metalloproteinase, estrogen and progesterone biosynthesis signaling, and inflammatory genes, making 12Z cells in monolayer comparable to human endometriotic tissue [36]; (5) 12Z cells molecularly respond to TNFα and IL6 signaling, both of which have been shown to be upregulated in the peritoneal fluid of women with endometriosis [40,41,42]; and (6) 12Z cells as small spheroids (~200 μm) better mimic human endometriosis lesions in histology and gene expression than monolayer [24]. While these previous works were imperative for moving the endometriotic field forward, we aimed to build bigger and more complex constructs assembled from multiple spheroids to better recapitulate endometriosis in women. To do so, we needed to develop building blocks of large spheroids (~500 μm) that could be biofabricated.

**Figure 2:**
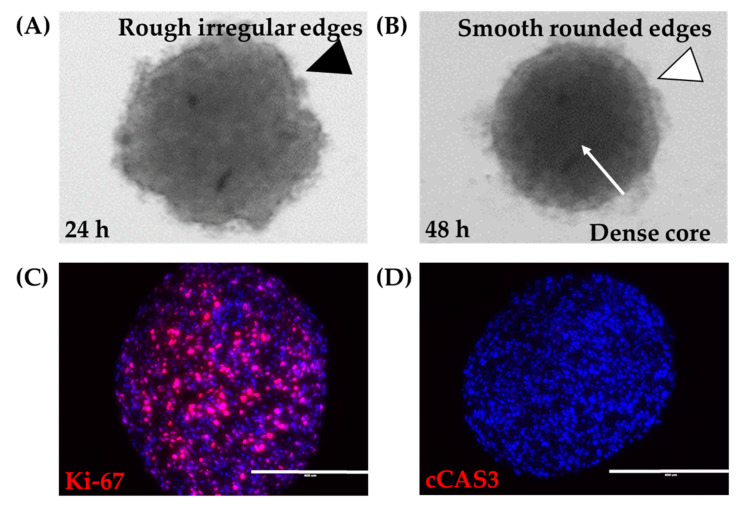
Large 12Z spheroids. (**A**) At 24 h, 18,000 12Z cells grown in ultra-low attachment round-bottom 96-well plates appear as a mass of cells with rough irregular edges (black arrowhead). (**B**) At 48 h, 12Z cells have formed a tight spheroid with a dense core (white arrow) and smooth rounded edges (white arrowhead). 12Z spheroids remain alive for at least 120 h, where the spheroids remained (**C**) proliferative (Ki-67, red) and (**D**) non-apoptotic (cCAS3, red). Blue = DAPI. Scale = 400 µm.

A spheroid was defined as a tight, dense mass of cells that, if disturbed by agitation or pipetting, the mass did not dissociate. 12Z cells grown in ultra-low attachment round-bottom plates were examined. By 4 h, cells were distributed evenly along the bottom of the wells (data not shown). At 24 h, cells had aggregated into a mass, with irregular tufts of cells on the mass’s outer edge (Figure 2A). By 48 h, cells had assembled into a tight, dense mass that maintained its solid structure upon agitation. Examination under bright field microscopy showed that the 12Z spheroids at 48 h were round in shape with smooth regular borders (Figure 2B). Since these spheroids were to be used as building blocks for larger constructs, there was a need to characterize their survival. Spheroids consumed significant amounts of nutrients, as evidenced by pH changes visualized through phenol red-containing media. Visual daily inspection showed no gross changes to spheroids after the initial formation. Proliferation was observed throughout the spheroid as indicated by evenly disbursed Ki-67 expression in sections cut from the center of the spheroid (Figure 2C). Spheroids remained without gross observations of media containing dead cells or spheroids breaking into smaller pieces. Additionally, cCAS3 was used to assess apoptosis. Spheroids were absent of apoptosis even at the core (Figure 2D). Thus, 12Z cells can be grown and maintained as large spheroids.

**Figure 3:**
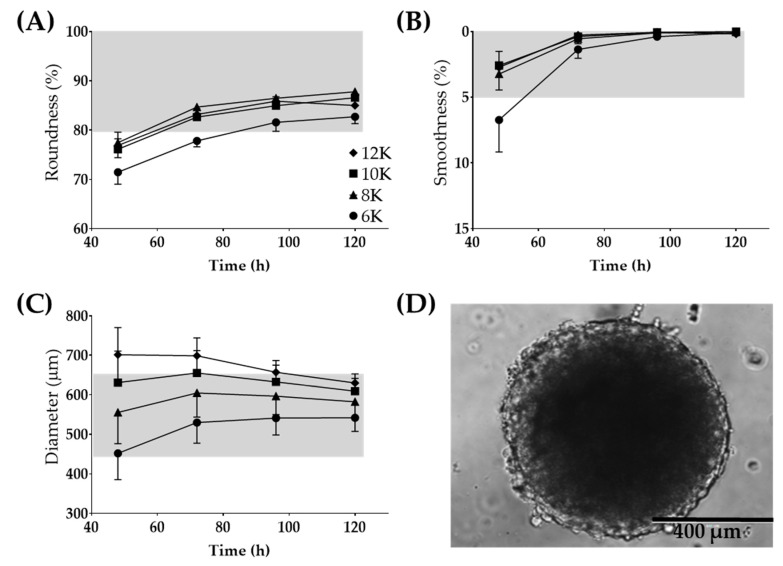
Large 12Z spheroids used for 3D biofabrication. Optimization for time post-seeding and the number of cells seeded for (**A**) roundness, (**B**) smoothness, and (**C**) diameter. (**D**) Representative image of a spheroid which fits all of the Regenova goal parameters. Scale = 400 µm. Grey shading indicates the tolerability range. N = 3.

The Regenova Bio 3D Printer uses spheroids as building blocks for more complex biofabricated tissue constructs [29] (Figure 1C). Specific spheroid characteristics need to be met before successful biofabrication. For example, the Kenzan is made up of needles spaced 500 µm apart. Spheroids that are too small will not fill in the gap between the needles, failing to fuse to other spheroids and thus failing to form a larger construct. Alternatively, too large of spheroids may not be placed on the Kenzan properly or may push adjacent spheroids out of place [30]. Thus, 500 µm spheroids are the optimal size with a tolerability range of 450–650 µm. The vision system was used to determine the spheroid roundness (%), smoothness (%), and diameter (µm) over 120 h when seeded at a density of 6000–12,000 cells per well (Figure 3). Consistent with gross visualization via bright field microscopy (Figure 2A), the Regenova Bio 3D Printer did not recognize the cells in the plate as spheroids at 24 h. Spheroids failed recognition by the Regenova Bio 3D Printer if they did not fit all of the user-defined optimal goal parameters [80–100 roundness (%), 0–5 smoothness (%), and 450–650 diameter (µm)]. In general, 12Z cell masses typically failed at 24 h because of unmet roundness or smoothness (data not shown). At 48 h, 12Z cells still failed to meet the optimal goal roundness (Figure 3A). By 72 h, most 12Z cells met optimal roundness and smoothness (grey shading in Figure 3A,B). Between 72 and 120 h, there was little or no change in roundness or smoothness. Time affected the diameter of spheroids (Figure 3C). As incubation time increased, the diameter converged on a range of 550–650 µm independent of cell density (Figure 3C). Low seeding density, 6000 cells per well, had consistently fewer round spheroids than all other groups, although, by 96 h, they met the optimal roundness criteria (Figure 3A). Seeding density did not appear to significantly impact the smoothness, except at 48 h (Figure 3B). At 72 h, spheroids made from 8000 12Z cells met all of the optimal goal standards and were used for further biofabrication (Figure 3D).

Previous work suggested that 12Z cells in monolayer were sensitive to serum changes [19]. Therefore, the effects of two different brands of United States-sourced FBS on 12Z spheroid roundness, smoothness, and diameter were examined. 12Z cells were grown in either Atlanta Biologicals or Corning FBS for two weeks before experiments. No gross differences in cell morphology, media consumption rate, or time to confluence in monolayer were observed (data not shown). Independent of which serum was used, at 72 h, 12Z cells resulted in tight, dense spheroids (data not shown). No sensitivity to serum brand was seen in either roundness or smoothness (Supplemental Figure S1A). However, spheroids increased in size, dependent on the number of cells seeded when generated in either serum (Supplemental Figure S1A). FBS from Atlanta Biologicals resulted in consistently larger spheroids when compared to spheroids generated in Corning FBS at 72 h (Supplemental Figure S1A). At 72 h, spheroids generated in Atlanta Biologicals serum ranged from 77–132 µm larger than spheroids generated in Corning serum with the same number of cells seeded. As a result, Atlanta Biologicals serum was used for the remainder of the experiments.

The final condition explored was the passage number of 12Z cells. Since 12Z cells were gifted at an unknown passage number, passage number was defined as early, middle, and late in reference to passaging within our laboratory. Passage number was relative to the number of continual passages since thawing, with early being < +5, middle = +5–15, and later > +15 passages. Roundness and smoothness did not appear to differ based on the passage number of the cells. 12Z cells that were passaged > +15 times and then cultured into spheroids tended to become larger, although not statistically significant (Supplemental Figure S1B). Table 1 gives the details of optimal conditions for 12Z cells for Kenzan biofabrication.

### 3.2. Characterization of 12Z Spheroids

**Figure 4:**
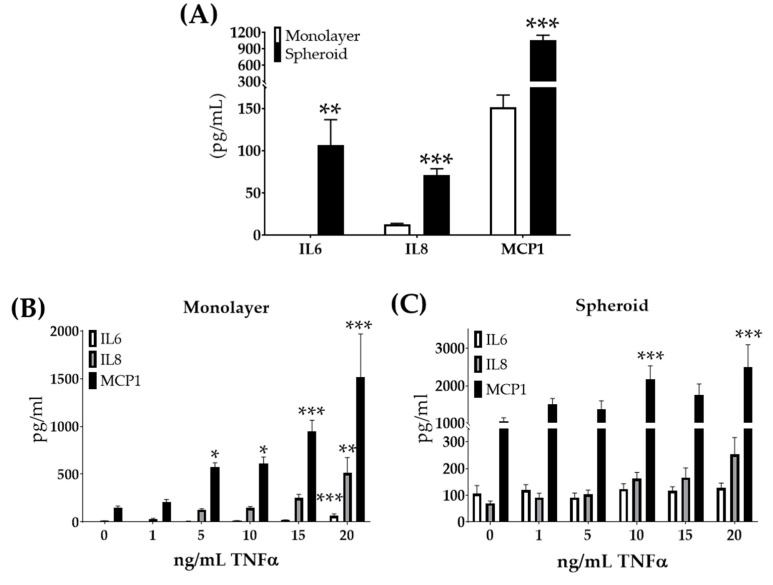
12Z cells secrete increased inflammatory factors when grown as large spheroids but lose further sensitivity to TNFα. (**A**) Secreted inflammatory factors are increased in 12Z cells grown as large spheroids compared to monolayer. (**B**) Monolayer cells treated with TNFα secrete increased levels of inflammatory cytokines in a dose-response manner. (**C**) 12Z spheroids do not similarly respond, with only MCP1 secretion increasing after high doses of TNFα. Note the scale differs between graphs. N ≥ 3. * *p* < 0.05, ** *p* < 0.01, *** *p* < 0.001.

In addition to meeting the Regenova Bio 3D Printer biofabrication parameters, these large 12Z cell spheroids needed to maintain properties consistent with the endometriosis. Previous work had shown increased expression of inflammatory response genes in endometriomas [43]. Further, the upregulation of inflammatory factors IL6, IL8, and MCP1 [44,45,46,47,48,49,50] has been found in the peritoneal fluid of women with endometriosis. To study if growth in spheroid culture altered these inflammatory factors’ secretion, 12Z spheroids were grown. The supernatants from 12Z monolayer and spheroid cultures with the same number of cells and conditions were compared. When grown as spheroids, 12Z cells secreted significantly more IL6, IL8, and MCP1 than monolayer (Figure 4A). Previous work showed that 12Z cells in monolayer respond molecularly to TNFα with increased gene expression of *IL6*, C-X-C motif chemokine ligand 8 (*CXCL8*, also referred to as IL8), and chemokine ligand 2 (*CCL2,* also referred to as MCP1) [40]. Thus, we studied the effects of TNFα stimulation on large 12Z spheroids. Similar to the previous studies [40], 12Z cells in monolayer treated with increasing doses of TNFα responded with the increased secretion of IL6, IL8, and MCP1 (Figure 4B). 12Z spheroids treated with TNFα did not show an increase in IL6 or IL8 secretion (Figure 4C). MCP1 secretion did increase in 12Z spheroids when treated with TNFα (Figure 4C). 12Z spheroids were insensitive to TNFα with regard to IL6 and IL8 expression, unlike the high response to TNFα observed in 12Z monolayer.

**Figure 5:**
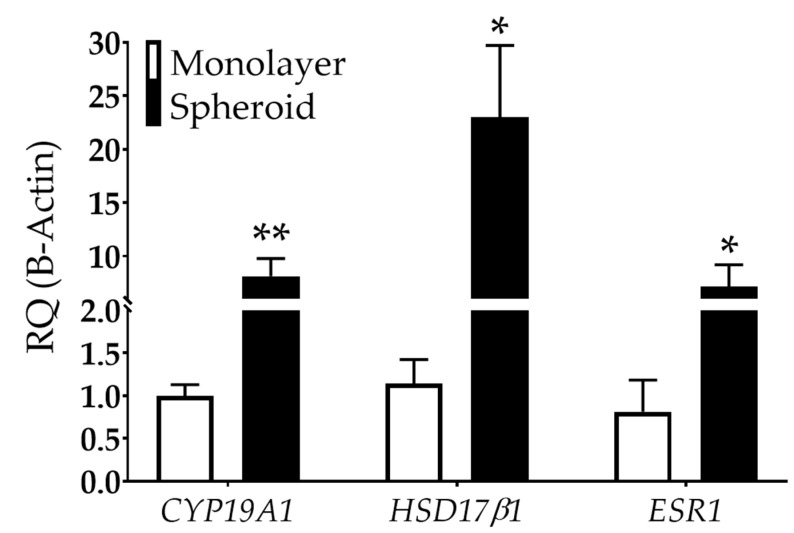
Estrogen-related gene expression is dependent on dimensionality in 12Z cells. *CYP19A1* and *HSD17β1*, genes involved in estrogen synthesis, are upregulated in 12Z cells grown as spheroids compared to monolayer. *ESR1*, an estrogen receptor, is also increased in spheroids compared to monolayer. RQ = relative quantification. N ≥ 3. *p < 0.05, ** *p* < 0.01.

Studies have also shown that endometriotic cells can metabolize estradiol, potentially exacerbating the disease [51,52]. This de novo estrogen synthesis may be caused by available cholesterol coupled with increased aromatase [cytochrome p450, family 19a1 (*CYP19A1*)] and hydroxysteroid 17-beta dehydrogenase 1 (*HSD17β1*) expression, critical enzymes involved in estradiol synthesis [53,54,55]. Large 12Z spheroids showed an 8.1-fold increase in *CYP19A1* and a 23-fold increase in *HSD17β1* in spheroid than monolayer cultures (Figure 5). Further, 12Z spheroids exhibited a 7.2-fold increase in estrogen receptor type 1 (*ESR1*) compared to monolayer (Figure 5).

### 3.3. 3D Biofabrication with 12Z Spheroids

**Figure 6:**
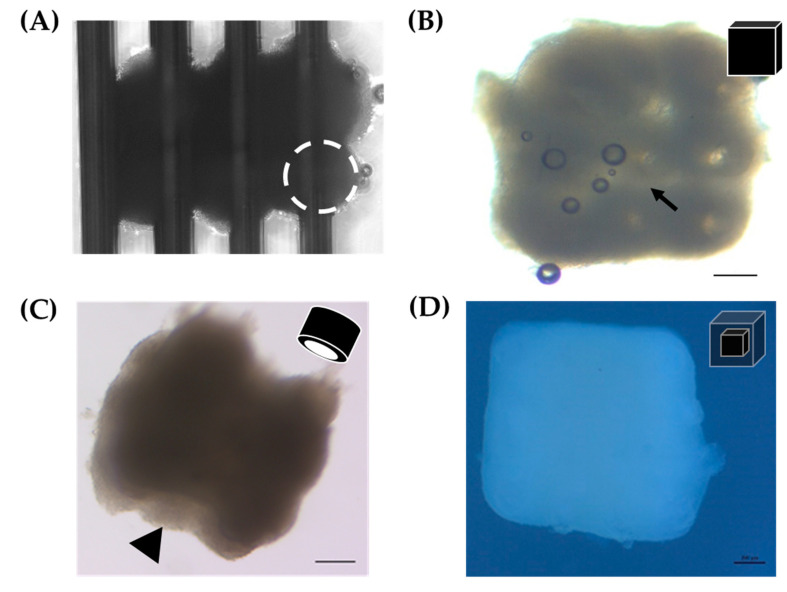
3D biofabrication of 12Z and HEYA8 spheroids into constructs. (**A**) 12Z spheroids immediately after biofabrication in a 3 × 3 × 2 pattern on the Kenzan. White dotted circle = individual spheroid. (**B**) Rendering and representative example of the 3 × 3 × 2 3D biofabricated cube made from 12Z spheroids < 24 h after removal from Kenzan. Scale = 200 µm. Arrow = hole left by Kenzan needle. (**C**) Rendering and representative example of a 3D printed tube made from 3 × 3 × 3 12Z spheroids > 24 h after removal from Kenzan. Scale = 200 µm. Arrowhead = hollow channel. (**D**) Rendering and representative construct from a 2 × 2 × 2 HEYA8 core surrounded by a 4 × 4 × 4 12Z shell. Scale = 200 µm.

3D biofabrication was performed on a 9 × 9 Kenzan. The Kenzan was used as a temporary scaffold allowing the spheroids to be held in position as they fuse to one another (Figure 1). With the robotic assistance of the Regenova Bio 3D Printer, 12Z spheroids (8000 cells per well, cultured for 72 h) were biofabricated by stacking three 12Z spheroids in the X and Y dimension and two spheroids in the Z dimension, 3 × 3 × 2 (~2.3 mm^3^, Figure 6A). Constructs were also generated from 2 × 2 × 2 (~1 mm^3^) and 3 × 3 × 3 (~3.4 mm^3^) spheroids (data not shown). After 72 h, the construct was gently lifted off the Kenzan by one of the two platens (Figure 1B). When constructs were first removed, they retained holes from where the Kenzan needles were (arrow, Figure 6B). These holes were no longer visible within 24 h, and borders between individual spheroids smoothed. The media was changed every 1–2 days. If constructs were placed in a traditional cell culture dish, they adhered to the dish floor, and cells would proliferate from the construct into monolayer (data not shown). Therefore, constructs were maintained in ultra-low attachment 12-well plates. Constructs were cultured for up to 96 h with no visual observations of construct degradation or breakage. To test the other shapes, 12Z spheroids were biofabricated into a hollow 3 × 3 × 3 tube (Figure 6C). Within 24 h after the construct was removed from the Kenzan, the needle holes had closed and were no longer visible, but the inner channel remained patent (arrowhead, Figure 6C).

### 3.4. Multi-Cell Type 3D Biofabrication

A significant portion of non-high-grade serous epithelial ovarian cancers, including endometrioid and clear-cell ovarian adenocarcinomas, are believed to be biologically impacted by the endometriotic microenvironment [12,56]. As a result, modeling endometriosis-associated ovarian cancers using 3D biofabrication techniques may allow for recapitulation of the endometriotic microenvironment. To study ovarian cancer within the endometriotic microenvironment, a malignant ovarian epithelial cell line, HEYA8, which was previously shown to grow as large dense aggregates [26], was selected. HEYA8 cells were optimized for spheroid formation compatible with 3D biofabrication (Supplemental Figure S2). HEYA8 cells did not show any sensitivity to serum changes (data not shown). Spheroids generated from 18,000 HEYA8 cells per well were used for biofabrication (Table 1).

Since 12Z cells and HEYA8 are typically maintained in different media (HEYA8, RPMI; 12Z, DMEM/F12), optimization for co-culturing was necessary. 12Z cells were labeled with Td-Tomato and HEYA8 cells with GFP for easy visual recognition under fluorescent microscopy. Fluorescently expressing cells were then grown together in monolayer with seven 12Z cells for every one HEYA8 cell, the final ratio of cells used in constructs is described below. The ratio of DMEM/F12 to RPMI media varied, and the relative cellular density of the two cell types was observed via fluorescent microscopy at 96 h (Supplemental Figure S3). The media ratio that best maintained the original seeding ratio (eight DMEM/F12: one RPMI) was used for constructs composed of 12Z and HEYA8 cells. Constructs were biofabricated using spheroids made from 12Z cells and spheroids made from HEYA8 cells. This construct was formed as a 2 × 2 × 2 HEYA8 (ovarian cancer) core surrounded by a 4 × 4 × 4 12Z (endometriosis epithelium) shell (Figure 6D). This multi cell-type 3D biofabricated construct was greater than 1.5 mm on each side and exhibited smooth edges.

### 3.5. 3D Biofabrication of Heterotypic Spheroids

Endometriotic lesions have cellular heterogeneity, being comprised of endometrial-like epithelial and stromal cells [1]. Consequently, having stromal cells within the same construct could better represent the endometriotic microenvironment. The T-HESC cell line [33] was chosen as the stromal cells for these experiments because (1) they are a human telomerase-immortalized benign endometrial stromal cell line, allowing for reproducible growth [33], (2) they are commercially available, (3) they have a comparable doubling time to 12Z cells, allowing for alignment of experimental logistic [33], (4) they have cellular, biological, and molecular properties of endometrial stromal cells [33], and (5) they are grown in similar media to 12Z cells, making T-HESC cells a promising stromal cell line to grow with 12Z cells. T-HESC cells were capable of forming tight, dense spheroids that withstood gentle disruption and fit within the Regenova Bio 3D Printer vision parameters for smoothness and diameter, but not roundness (Supplemental Figure S4A–C). Additionally, these spheroids failed to successfully biofabricate on a Kenzan in two ways. One, some spheroids failed to withstand the suction pressure and were deformed (Supplemental Figure S4D) or were sucked into the waste container, independent of pressure or gauge optimization. Two, spheroids that survived the suction pressure lost integrity upon contact with the Kenzan, leaving only partial spheroid remains, not capable of fusing into constructs (Supplemental Figure S4E).

**Figure 7:**
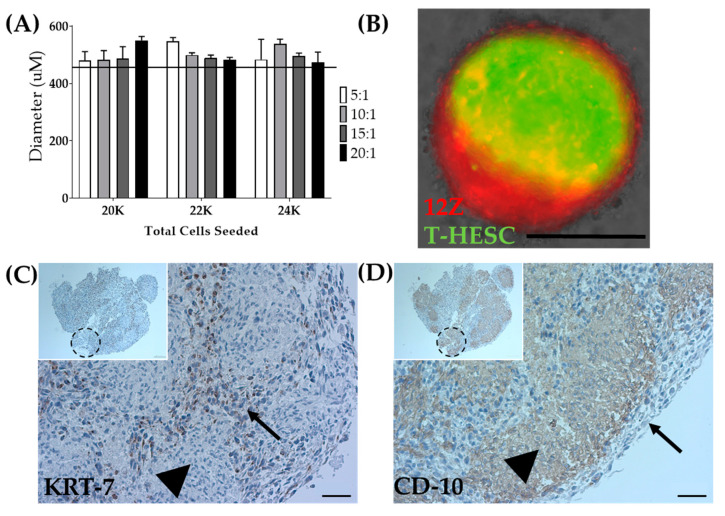
Biofabrication of heterotypic spheroids. (**A**) Optimization of diameter for the total number of seeded cells and ratio of T-HESC to 12Z cells at 72 h post-seeding. The line indicates the tolerability range of Regenova technology. N = 3. (**B**) Spheroids made from 12Z and T-HESC cells self-assembled with 12Z-epithelial cells (Red) on the outside and T-HESC-stromal cells (Green) on the inside. Scale = 400 µm. 3 × 3 × 3 constructs biofabricated from heterotypic spheroids share similar self-assembly with (**C**) KRT-7 as an epithelial marker and (**D**) CD-10 as a stromal marker. Arrow = epithelial cells (12Z), arrowhead = stromal cells (T-HESC), dashed circle = presumed location of an individual spheroid. Scale = 50 µm. Inset Scale = 100 µm.

As a means to overcome the inability to biofabricate T-HESC spheroids alone, T-HESC cells were co-cultured with 12Z cells, creating a heterotypic spheroid. T-HESC cells were marked with GFP, and 12Z cells were labeled with Td-tomato to discern cell types. Total cell number, the ratio of epithelial to stromal cells, and the time in culture were varied to determine the optimal spheroid formation conditions (Figure 7A). Heterotypic spheroids made from equal ratios of T-HESC and 12Z cells or a 5:1 ratio failed to survive the pressures of biofabrication. Ratios of 10:1, 15:1, and 20:1 were successfully biofabricated (data not shown). For biofabrication of heterotypic spheroids, spheroids were generated from a total of 22,000 cells seeded in a 10:1 (T-HESC:12Z) ratio at 72 h post-seeding (Table 1). Fluorescent tagging of both cell lines allowed for observation of the intra-spheroidal organization of the two cell types. The fixation protocol was optimized to minimize the cellular damage and fluorescent quenching (data not shown). Whole spheroid imaging showed that cells self-organize within the spheroid with 12Z cells creating a shell around a T-HESC core (Figure 7B), as observed in endometrial organoids [57].

Although cellular arrangement within the heterotypic spheroid appeared to self-assemble in a biologically relevant manner, cellular arrangement within the larger construct required testing. Following successful biofabrication of spheroids into a 3 × 3 × 3 construct, the constructs were fixed. Immunohistochemistry was used to confirm the self-organization in the constructs. Consistent with the fluorescent markers, KRT7, an epithelial marker, outlined the individual spheroids within the larger construct (Figure 7C), and CD10, a stromal marker, was found inside each individual spheroid (Figure 7D) [58,59]. Accordingly, both spheroids and constructs can be generated, including stromal and epithelial cell types distributed in a biologically relevant manner.

## 4. Discussion

Endometriosis research is hindered by the scarcity of representative endometriotic in vitro models [16,17,18,19]. Our study shows that large spheroids generated from 12Z cells represent specific human endometriosis characteristics better than monolayer culture. Additionally, we show that complex 3D tissue-like constructs can be successfully biofabricated to model endometriosis and the endometriotic microenvironment. In our experiments, 12Z spheroids were biofabricated into 3D constructs, and complexity was added by including ovarian cancer and stromal cells.

Despite a large number of ovarian cancer cell lines, there are a few benign gynecological cell lines modeling diseases such as endometriosis, and even fewer epithelial-like endometriotic cell lines [16,17,18,19]. Therefore, we chose to focus on a commercially available immortalized endometriotic epithelial-like cell line, 12Z, as the epithelial cell for these proof-of-concept models. 12Z has become one of the best characterized endometriotic cell lines, leading to molecular and functional insights into endometriosis [21,24,36,40,41,42,59,60,61]. For example, Arosh et al. used 12Z cells cultured with and without endometriotic stromal cells to highlight the molecular and preclinical impact of prostaglandin E_2_ in endometriosis [60]. Wu et al. used 12Z cells with long-term exposure to TNFα to explain the mechanism behind progesterone resistance in endometriotic lesions [42]. Some consider 12Z cells to have properties of benign endometrial cells [62]. We consider 12Z cells in monolayer a reasonable endometriosis model because 12Z cells express estrogen-related [36] and inflammatory genes [40].

For our work shown here, large 12Z spheroids represented an in vitro model of endometriosis in two biologically impactful ways. One, 12Z spheroids secreted increased inflammatory factors, including IL6, IL8, and MCP1, compared to monolayer, which recapitulates what is seen in women with endometriosis [63,64]. Inflammatory dysregulation, such as increased TNFα and transforming growth factor-beta signaling, plays a vital role in endometriosis’s pathogenesis [17,43,65,66]. Inflammatory cytokines are elevated in endometriosis lesions and peritoneal fluid of women with endometriosis [44,45,46,47,48,49,50]. Specifically, elevated IL8 and MCP1 have been shown in the peritoneal fluid of women with endometriosis [64], and IL6, in the peritoneal fluid, has been linked to infertility in women with endometriosis [63]. Thus, the recapitulation of inflammatory dysregulation is a necessary characteristic of a robust in vitro model. Grund et al. previously showed that 12Z cells stimulated with TNFα increased inflammatory signaling [40]. We showed that 12Z cells in monolayer express undetectable or low levels of inflammatory cytokines, but similar to Grund et al. [40], TNFα stimulation increased the secretion of IL6, IL8, and MCP1. This data suggest that monolayer 12Z cells require outside stimuli to represent the inflammatory microenvironment seen in women with endometriosis.

In contrast, we showed that 12Z cells could reproduce the robust inflammatory signaling without the need for additional stimulation if they are grown as large 3D spheroids. Our work using secreted cytokines supports gene expression data from small 12Z spheroids previously reported [24]. We further challenged this robust inflammatory phenotype by treating spheroids with TNFα, where neither IL6 nor IL8 responded. Interestingly, MCP1 did increase in response to TNFα treatment. This dichotomous response may indicate that IL6 and IL8 are induced through separate mechanisms from MCP1 in endometriosis. It has been suggested that through non-genomic pathways, increased estrogen signaling in endometriosis can stimulate MCP1 [67]. Therefore, one hypothesis suggests that increased estrogen signaling in 12Z spheroids may be driving the MCP1 secretion, allowing for TNFα stimulation to exacerbate the secretion. Additional hypotheses could offer other TNFα-independent pathways or that IL6 and IL8 reached a ceiling, and thus, no further response after TNFα stimulation was possible. Further testing is needed to explore this effect. Nevertheless, 12Z spheroids can be used to model endometriosis-related inflammation with less need for cellular manipulation and may provide fascinating insights into the pathogenesis of endometriosis-related inflammation.

Two, the 12Z in vitro spheroid model represented endometriosis with elevated levels of estrogen-related genes: *CYP19A1*, *HSD17β1*, and *ESR1*. These data suggest that 3D biofabricated constructs may be a good representation of human disease. Current medical therapies for endometriosis target the reduction of estradiol levels since endometriosis is an estrogen-responsive disease [2]. Both endometriotic lesions and 12Z cells have been shown to express ESR1, allowing estrogen response [21,68]. Here, we showed that *ESR1* gene expression was significantly increased when 12Z cells were grown as spheroids. This result suggests that 12Z spheroids are more estrogen-responsive, although additional testing will need to be completed. Studies indicate that endometriotic lesions themselves may produce exogenous estrogen through available cholesterol and increased aromatase [68]. We showed that 12Z spheroids have an increase in *CYP19A1*, which was previously reported in small 12Z spheroids [24]. *HSD17β1* encodes for the enzyme responsible for estrone conversion to the bio-actively available 17β-estradiol. *HSD17β1* is expressed in 12Z monolayer cells [36] but is expressed at increased levels in 3D spheroids. In total, we showed that culturing 12Z cells into large spheroids significantly stimulates the expression of genes involved in estrogen synthesis, suggesting that 12Z 3D spheroids can be used to study the exogenous expression of estrogen in endometriosis. Although de novo production of estrogen in spheroids recapitulates one aspect of the hormone milieu, endometriosis is an estrogen-responsive disease [2] which necessitates future experiments that assess the impact of hormone stimulation on spheroids. A subset of spheroids was maintained for 18 days with no discernable gross signs of death, suggesting spheroids can be grown long-term (data not shown). This extended growth suggests that studying the impact of hormone stimulation on gene expression, inflammation, and morphology is feasible.

The reasons listed here support the use of 12Z cells as an in vitro model of endometriosis, albeit 12Z cells impose limitations since they do not reflect the entire biologic complexity of endometriosis. For example, 12Z cells were derived from a red peritoneal lesion [21]. Studies have shown unique molecular profiles between different anatomically locations of endometriosis, including rectovaginal, peritoneal, and ovarian endometriomas [69]. Further, eutopic endometrium and ectopic endometriosis from the same woman are molecularly distinct [70]. Thus, the use of a single immortalized cell line such as 12Z cannot be extrapolated to all distinct molecular forms of endometriosis. Future studies are needed to develop novel endometriotic cell lines [18,19]. Based on the limited number of cell lines used in our proof-of-concept studies, these studies are limited by the in vitro nature and the biologic ability of each cell line to only partially recapitulate the intricate features of endometriosis.

While spheroid models do pose a better way to in vitro model human disease, limitations like the inability to recapitulate a tumor’s complexity because of the small size and lack of microenvironment interactions still remain [71]. Therefore, we used the Kenzan method of biofabrication to increase both the size and complexity of endometriotic in vitro models. The Kenzan method allows for larger constructs by using spheroids as building blocks. One limitation of the Kenzan method is the reliance on the cell line of interest to form spheroids compatible with the Regenova standards. Here we showed 12Z cells were sensitive to changes in serum as previously published [19], which resulted in spheroids that failed to consistently print due to the nozzle failing to lift the spheroids (Supplemental Figure S1). This phenomenon suggests that careful optimization is necessary. Additionally, we showed that T-HESC cells alone do not withstand the pressures of the nozzle, although this was overcome by growing them in a heterotypic spheroid with 12Z cells. With proper optimization of compatible cell lines, as shown here, we increased the size of the in vitro model to 27X the original spheroid size. However, we do not yet know the optimal size for constructs. In addition to allowing for increased size, the Kenzan method supports highly reproducible and versatile constructs, allowing for better recapitulation of tissue interaction and biologically relevant shapes [31]. The Regenova Bio 3D Printer’s robotic arm allows for precise placement in the X, Y, and Z dimensions on the Kenzan, increasing reproducibility. We showed 12Z spheroids could be biofabricated into multiple shapes and sizes, including cubes and hollow tubes. Our dense cube constructs can be used to model tumors, while the tube structures can be used to study perfusion and disseminate cells. These results represent proof-of-concept data that the Kenzan method can produce scaffold-free constructs made from an endometriotic cell line. These constructs may better recapitulate endometriotic cell responses to environmental or treatment stimulation. Currently, the Regenova Bio 3D Printer can print tissue-like constructs ranging in size from one spheroid to a 10 mm × 10 mm × 10 mm cube. The other important limitation is that the final structure must be handled delicately and aseptically at all times. Finally, previous work has shown that these constructs can be perfused, allowing the ability to study steroid hormone effects but also the effects of laminar flow [72].

Biofabricated constructs not only allow for larger size and more shapes but can be used to model the microenvironment and cellular interactions. Here, novel in vitro modeling of the endometriotic microenvironment has been demonstrated by generating complex 3D biofabricated tissue-like constructs. Lee et al. previously described the ability for many different epithelial ovarian cell lines to form or fail to form spheroids [26]. This body of work was substantial and beneficial for the early development of 3D cell cultures to model ovarian disease. It was used here to choose a malignant cell line to biofabricate into constructs. The HEYA8 cell line formed tight, dense spheroids [26] and has been well characterized molecularly as non-high-grade serous [73,74]. To create an in vitro model of the endometriotic microenvironment, we biofabricated multi-cell type 3D constructs with HEYA8 and 12Z spheroids. Specifically, multi-cell type 3D constructs were biofabricated from a core of malignant HEYA8 cells surrounded by a shell of benign 12Z cells. A particular limitation to this model is that 12Z cells are derived from peritoneal lesions [21], while most but not all endometriosis-associated ovarian cancers develop from ovarian endometriomas [12,13,14,15]. To our knowledge, this is the first Kenzan construct formed from two distinct monotypic spheroids. This multi-cell type construct is a proof-of-concept that the endometriotic microenvironment can be modeled through the Kenzan method. Further investigation of the intercellular interactions may provide insights into how benign and malignant tissue impact one another. Confirmation of cellular organization, invasion, migration, and fusion across cell types should be examined to investigate the natural dispersion after culturing together. While we used the Kenzan method to model the endometriotic microenvironment, this method may be used for a much broader study of gynecological disorders and normal tissue. The Kenzan method is limited by the ability of cell lines to be grown into compatible spheroids within the system requirements (i.e., roundness, smoothness, diameter), but if these are met, a wide range of constructs can be developed both in and out of gynecological sciences. For instance, this methodology with multiple cell types under perfusion may be impactful in the development of an in vitro model of normal endometrium.

Complex 3D in vitro models have previously been developed and are used for gynecological studies in endometrial and ovarian regeneration [75,76,77,78,79] and cervical remodeling [80,81,82]. For instance, to recapitulate the hypothalamic-pituitary-ovarian axis, researchers have built a microfluidic system using a chemical scaffold material [77]. Unfortunately, scaffold materials can impose severe limitations on cellular diversity, viability, and intercellular communication [71,83]. Specifically, naturally or synthetically derived scaffold materials tend to prevent normal interactions causing poor replication of normal tissue. Natural tissue development utilizes input from both cell–cell and cell–extracellular matrix interactions to determine the proper nutrient and waste transfer [71,83,84]. These biologic requirements pose a challenge for engineering endometriosis and endometriotic microenvironment tissue models. The Kenzan method [29] allows for the production of scaffold-free tissue-like constructs. For biofabrication, the Kenzan is used as a temporary scaffold, relying on cells to deposit native, biologically optimized extracellular matrix. The constructs are then removed from the Kenzan, leaving a fully scaffold-free construct. Here, we showed the robust potential of the Kenzan method to produce scaffold-free tissue-like constructs.

Finally, endometriosis and the endometriotic microenvironment are challenging to investigate because of the high complexity of the intercellular communication between unique cell types, including endometrial epithelium and stroma. To better represent the endometriotic tissue in vitro, the complexity of 3D biofabricated tissue-like constructs was increased using heterotypic spheroids generated from T-HESC and 12Z cells. 3D modeling of endometriosis has previously been established using long-term expandable organoid models from primary tissue that recapitulated disease traits and common mutations [78,85]. But primary cell models pose a risk of limited reproducibly due to the limited tissue availability. Both the 12Z and T-HESC cells are immortalized and commercially available, resulting in increased reproducibility and availability. Also, 12Z [21] and T-HESC [33] cells have a relatively quick doubling time allowing for sufficient cell density to generate spheroids, and spheroids can be generated in 72 h. Additionally, while organoid models recapitulate some disease traits, they are constrained to a relatively small size [78,85] or can take weeks to months to form large organoids [86]. Here, we show that spheroids can be used as building blocks to generate larger constructs, and 72 h was sufficient time on the Kenzan for spheroids to fuse into larger tissue constructs. Therefore, time from monolayer to fully formed large scaffold-free tissue constructs (Figure 6) is less than one week. For the scope of this study, we highlight the least amount of time necessary to generate spheroids and constructs of interest, but limitations due to maximum time were not assessed. Previously, constructs generated using the Kenzan method have been cultured for up to two weeks, with reports of high integrity [31]. Thus, we believe it is feasible to culture these constructs for longer periods of time to assess the morphological changes in a time-dependent manner or allow for treatment or stimulation of the construct.

Endometriosis is pathologically composed of both epithelial and stromal cells. To bring this biologic relevance to our model, T-HESC cells were added to 12Z cells in heterotypic spheroids. T-HESC cells are considered a normal endometrial stromal cell line and not endometriotic, allowing for the unique ability to study the epithelium’s role in endometriosis pathogenesis. Endometriotic stromal cells can and should be supplemented for T-HESC in the future to explore the part of the endometriotic stromal cells in the pathogenesis of endometriosis. T-HESC cells were successfully generated as monotypic spheroids but failed to meet the Regenova Bio 3D Printer standards and the pressures of the suction nozzle during biofabrication. T-HESC cells may produce less extracellular matrix naturally, thus providing less stability to the overall structure. Our goal is to have biologically relevant cellular interactions. We optimized heterotypic spheroids, spheroids composed of both epithelial cells and stromal cells. Excitingly, our findings indicate that the heterotypic spheroids self-assembled in a biologically meaningful way [57]. At seeding, the cells were a homogenous mixture of T-HESC and 12Z cells. After spheroid formation, T-HESC cells had condensed in the center while being surrounded by a 12Z shell. When spheroids were biofabricated into larger constructs, they maintained their core and shell pattern. However, longer-term culturing may result in epithelial cells from individual spheroids migrating out to the construct boundaries. Experiments analyzing the long-term culturing capabilities and the cellular distribution over time would bring significant insight into the dynamic capabilities of constructs. Additionally, we did not histologically see any glandular or tubular formation. Others have shown that injection of 12Z cells with the immortalized endometriotic stromal cell line 22B in a 1:1 ratio using Matrigel gives glandular histology in an immunocompromised mouse model supplemented with estradiol after 30 days [60,87]. Finally, organoids of eutopic endometrium or ectopic endometriosis exhibited the morphology of glandular epithelium after being grown in Matrigel with estradiol for 14 days [78,85]. Thus, we envision that glandular morphology will require stimulation with steroid hormones or additional time in culture, whereas we only grew 3D-biofabricated constructs for 24 h off Kenzan. Additional histological limitations include the lack of pro-fibrotic phenotype in the relatively short-term cultures of 3D-biofabricated models. Recently proposed changes to the pathological definition of the ovarian cyst of endometriosis suggest that fibrosis must be present, along with epithelium and stroma, and that fibrosis is derived from transdifferentiation of cells, normally present within the ovary, such as myofibroblasts [88]. Our future studies will focus on both steroid hormone stimulation, mechanisms of cellular transdifferentiation, and fibrosis deposition. While this is not the first construct biofabricated using the Kenzan method from heterotypic spheroids [89], to our knowledge, it is the first using immortalized cells and modeling gynecological disorders.

## 5. Conclusions

In conclusion, we have shown that 12Z cells can be grown as large spheroids, capable of surviving Kenzan method of 3D biofabrication. These large spheroids represent the inflammatory and estrogen-related gene expression of endometriosis. These large 12Z spheroids can be used as building blocks to generate representative tissue-like 3D biofabricated models of endometriosis and the endometriotic microenvironment. We have provided proof-of-concept constructs that may lead to more complex, larger constructs of endometriotic in vitro models. Specifically, we have demonstrated the ability to culture constructs in biologically relevant shapes, dense cubes modeling tumors and tubes modeling perfused tissues, and demonstrated constructs with multiple cell types to study cellular interactions.

## Figures and Tables

**Table 1:** Biofabrication conditions for cell lines.

	12Z	HEYA8	12Z:T-HESC
**Number of Cells/Well**	8000	18,000	2000:19,000
**Medium**	DMEM:F12 + 10% Atlanta Biologicals FBS + 1% P/S	RPMI 1640 + 10% Atlanta Biologicals FBS + 1% P/S	DMEM:F12 + 10% Atlanta Biologicals FBS + 1% P/S
**Time in Culture (Hours)**	72	72	72
**Nozzle (Gauge)**	26	26	26
**Nozzle Pressure (kPa)**	2	2	2

## Supplementary Materials

The following are available online at https://www.mdpi.com/2227-9059/8/11/525/s1. Figure S1: 12Z cell spheroids are sensitive to changes in serum. Figure S2: Optimization of time and number of seeded cells for the spheroid formation and successful biofabrication of HEYA8 cells. Figure S3: Co-culturing cells in monolayer to determine the optimal media. Figure S4: T-HESC cells formed spheroids but failed to meet all of the Regenova Bio 3D Printer criteria for biofabrication.
