# Supplementary material for: Three-Dimensional Biofabrication Models of Endometriosis and the Endometriotic Microenvironment
Source: Biomedicines. 2020 Nov 21;8(11):525. doi: 10.3390/biomedicines8110525 (PMC7700676; doi:10.3390/biomedicines8110525)
Supplement: Supplementary file 1 [file biomedicines-08-00525-s001.pdf]

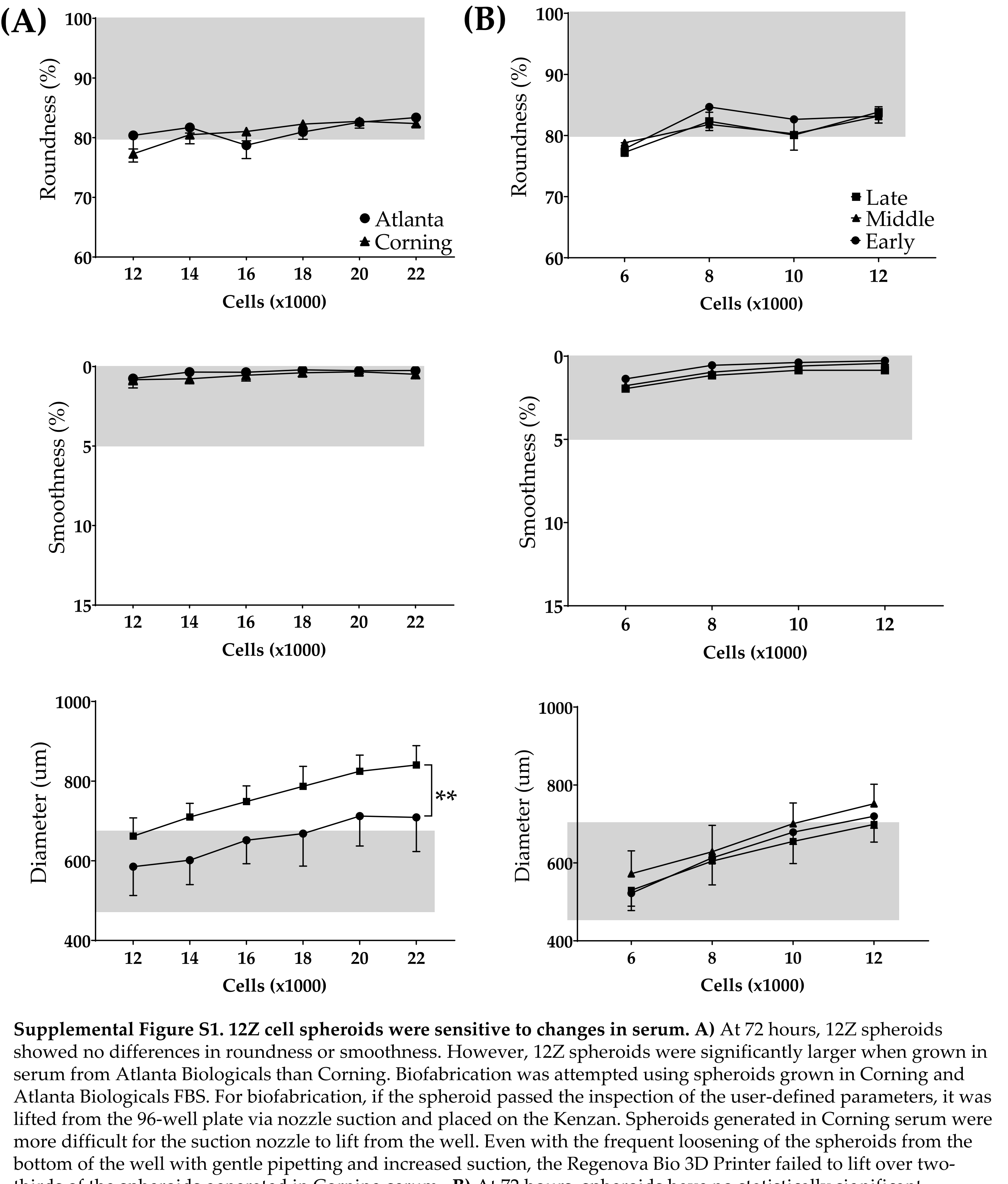

Supplemental Figure S1. 12Z cell spheroids were sensitive to changes in serum. A) At 72 hours, 12Z spheroids showed no differences in roundness or smoothness. However, 12Z spheroids were significantly larger when grown in serum from Atlanta Biologicals than Corning. Biofabrication was attempted using spheroids grown in Corning and Atlanta Biologicals FBS. For biofabrication, if the spheroid passed the inspection of the user-defined parameters, it was lifted from the 96-well plate via nozzle suction and placed on the Kenzan. Spheroids generated in Corning serum were more difficult for the suction nozzle to lift from the well. Even with the frequent loosening of the spheroids from the bottom of the well with gentle pipetting and increased suction, the Regenova Bio 3D Printer failed to lift over two-thirds of the spheroids generated in Corning serum. **B)** At 72 hours, spheroids have no statistically significant differences in roundness, smoothness, or diameter as passage number increases. Since spheroid generation occurred in all passage numbers of 12Z cells, biofabrication was attempted. During biofabrication attempts, it was noted that > +15 passages resulted in spheroids that failed to be lifted by the nozzle. Like the Corning serum, spheroids generated from > +15 passaged cells failed to be raised by the suction nozzle even after significant optimization of nozzle gauge and suction pressure. As a result, 12Z cells from ≤ +15 passages were used for biofabrication. Early passage was < +5, middle = +5-+15, and late < +15. N = 3. **, *P* < 0.01.

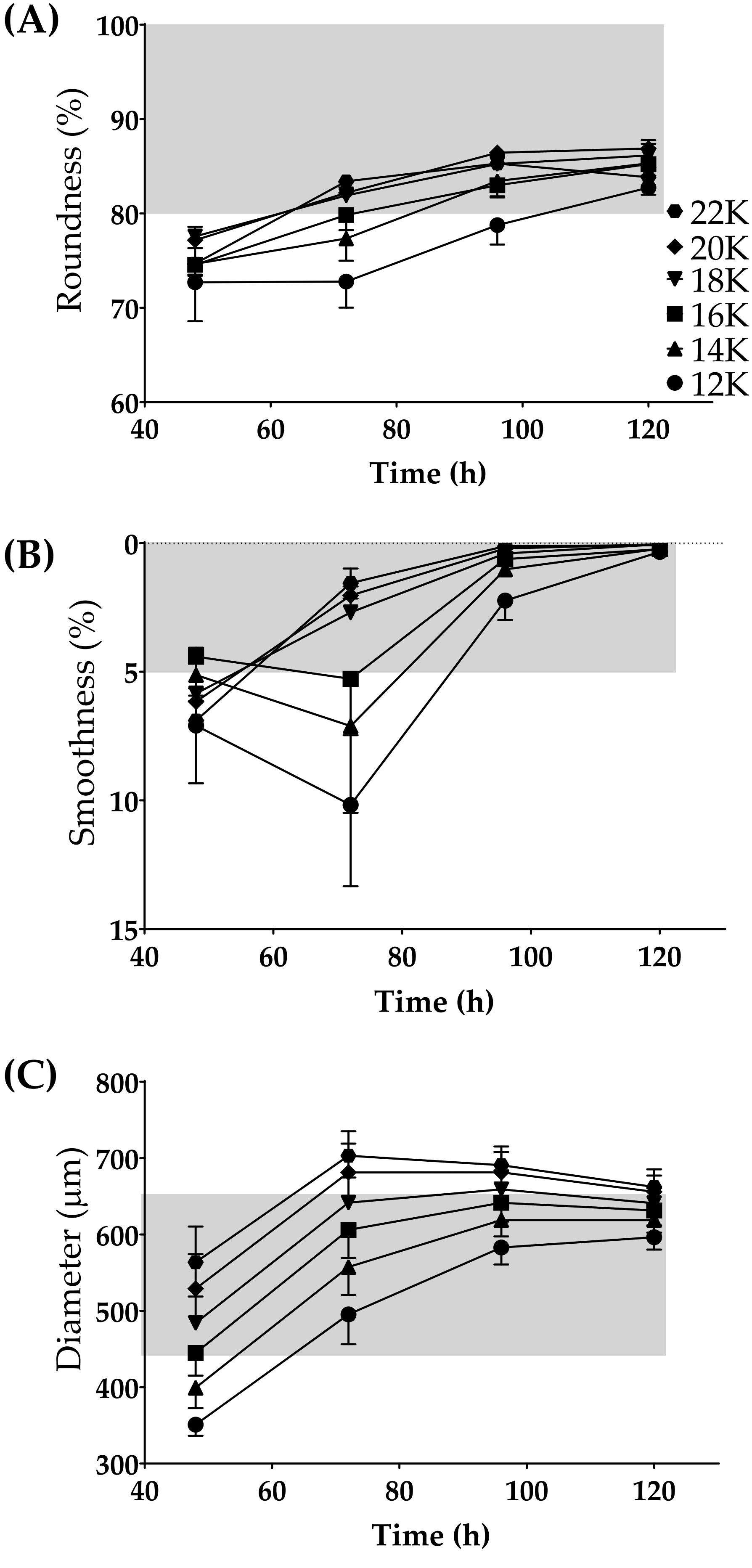

Supplemental Figure S2. HEYA8 spheroid characteristics. Optimization for time post-seeding and number of cells seeded for **A)** roundness, **B)** smoothness, and **C)** diameter. Grey shading indicates tolerability range of Regenova technology. N = 3.

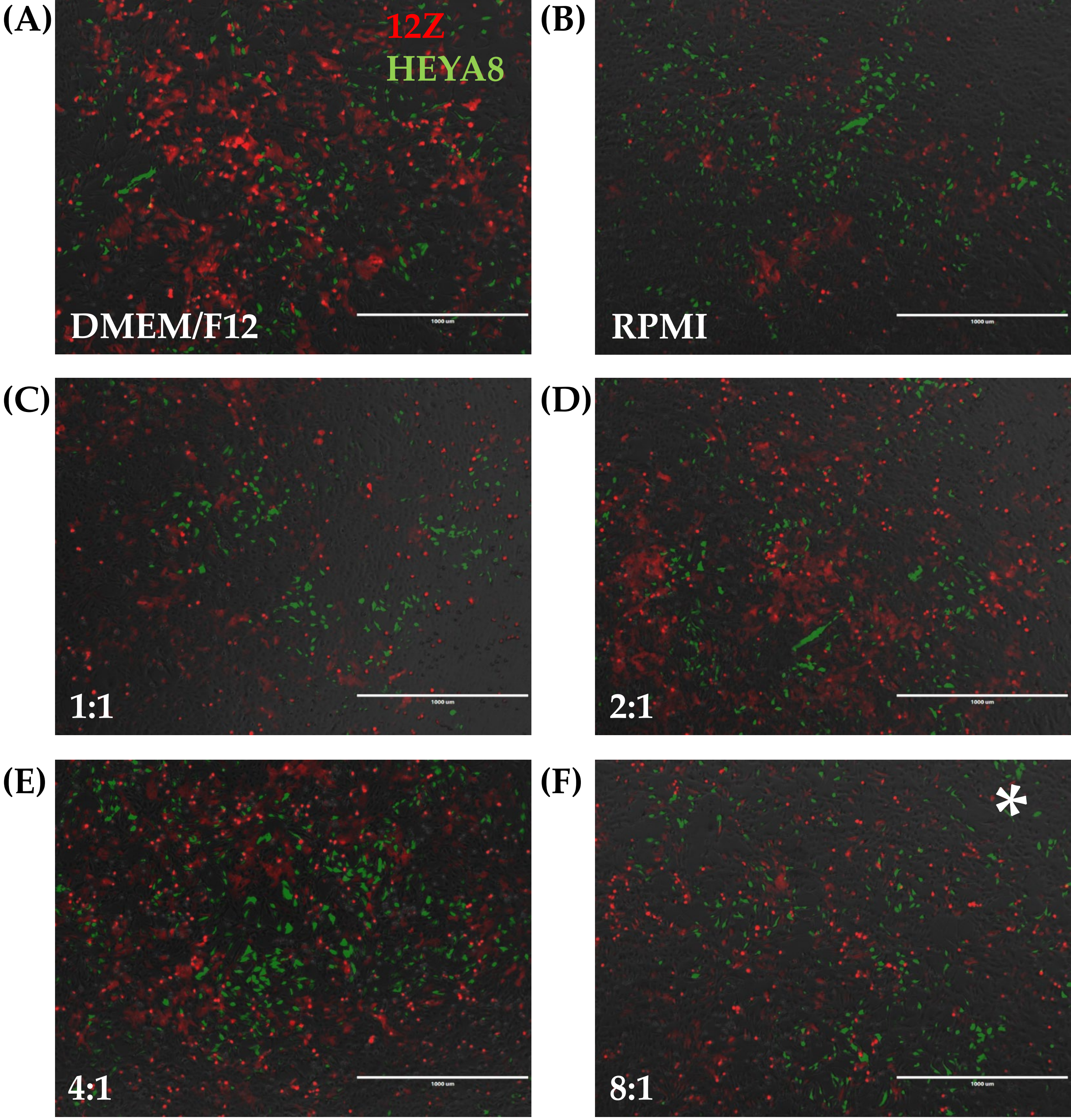

Supplemental Figure S3. Co-culturing cells in monolayer to determine optimal media. Cells were cultured for 96 hours in a 6-well plate with a total of 100,000 cells at a 7-12Z:1-HEYA8 ratio. Media used was **A)** DMEM/F12, **B)** RPMI, **C)** 1-DMEM/F12:1-RPMI, **D)** 2-DMEM/F12:1-RPMI, **E)** 4-DMEM/F12:1-RPMI, or **F)** 8-DMEM/F12:1-RPMI. Scale = 1000 μm. * Indicates media that appropriately supports growth of both cell lines in co-culture.

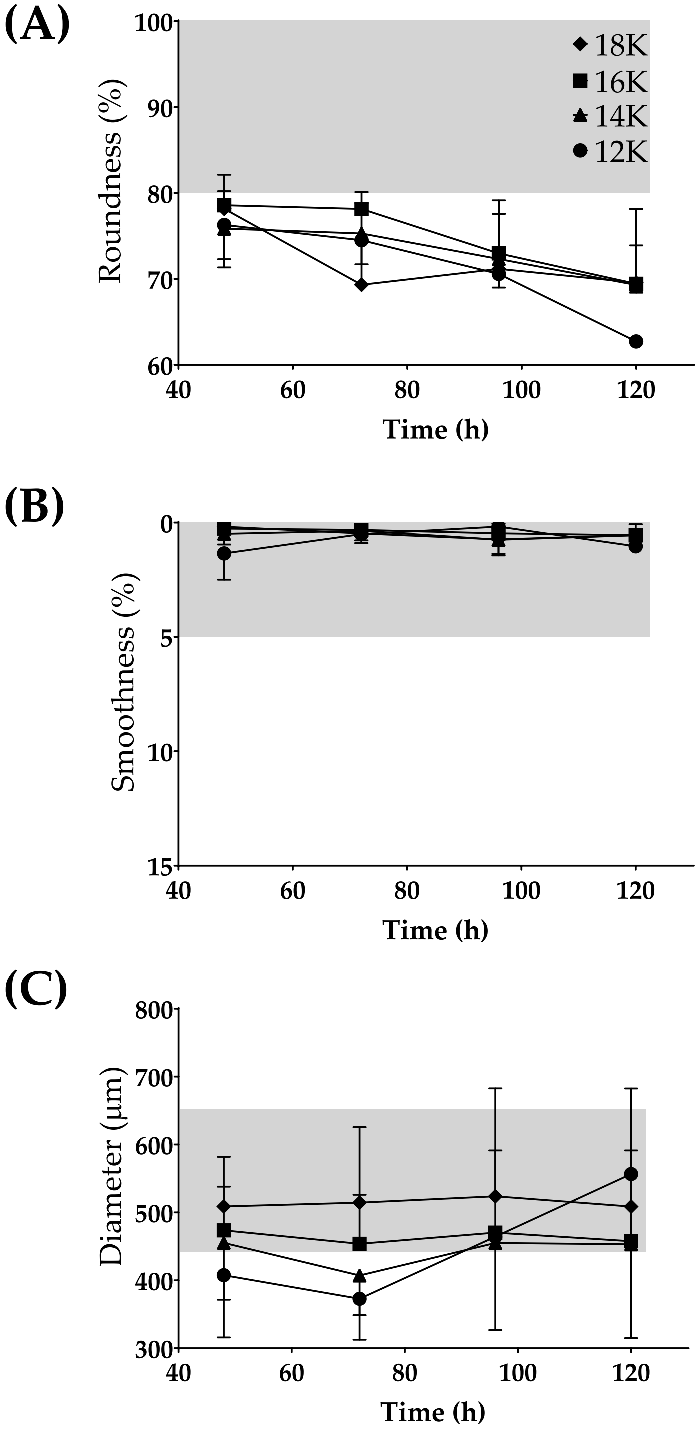

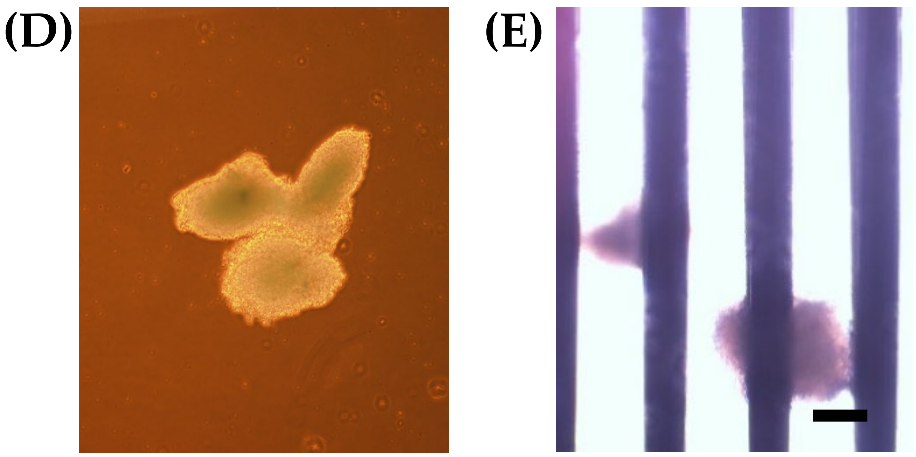

Supplemental Figure S4. T-HESC cells formed spheroids but failed to meet all of the Regenova Bio 3D Printer criteria for biofabrication. Optimization for time post-seeding and number of cells seeded for **A)** roundness, **B)** smoothness, and **C)** diameter. Grey shading indicates tolerability range of Regenova technology. N= 3. **D)** Spheroids made from T-HESC cells alone become misshapen from the suction or **E)** shear on contact with the Kenzan. Scale = 200 µm.
